# The effect of systemic iron status on osteoarthritis: A mendelian randomization study

**DOI:** 10.3389/fgene.2023.1122955

**Published:** 2023-03-16

**Authors:** Guangfeng Ruan, Yi Ying, Shilong Lu, Zhaohua Zhu, Shibo Chen, Muhui Zeng, Ming Lu, Song Xue, Jianwei Zhu, Peihua Cao, Tianyu Chen, Xiaoshuai Wang, Shengfa Li, Jia Li, Yu Liu, Yanqi Liu, Yan Zhang, Changhai Ding

**Affiliations:** ^1^ Clinical Research Centre, Guangzhou First People’s Hospital, School of Medicine, South China University of Technology, Guangzhou, China; ^2^ Department of Hematopathology, Guangzhou First People’s Hospital, School of Medicine, South China University of Technology, Guangzhou, China; ^3^ Department of Imaging Diagnosis, Zhujiang Hospital, Southern Medical University, Guangzhou, China; ^4^ Clinical Research Centre, Zhujiang Hospital, Southern Medical University, Guangzhou, China; ^5^ Department of Immunology, School of Basic Medical Sciences, Anhui Medical University, Hefei, China; ^6^ Department of Rheumatology and Immunology, Arthritis Research Institute, The First Affiliated Hospital of Anhui Medical University, Hefei, China; ^7^ Department of Orthopedics, Guangzhou First People’s Hospital, School of Medicine, South China University of Technology, Guangzhou, China; ^8^ Menzies Institute for Medical Research, University of Tasmania, Hobart, TAS, Australia

**Keywords:** iron, rs1800562, hip osteoarthritis (hip OA), total hip replacement (THR), mendelian randomization (MR)

## Abstract

**Objective:** To assess the causal effect of systemic iron status by using four biomarkers (serum iron; transferrin saturation; ferritin; total iron-binding capacity) on knee osteoarthritis (OA), hip OA, total knee replacement, and total hip replacement using 2-sample Mendelian randomization (MR) design.

**Methods:** Three instrument sets were used to construct the genetic instruments for the iron status: Liberal instruments (variants associated with one of the iron biomarkers), sensitivity instruments (liberal instruments exclude variants associated with potential confounders), and conservative instruments (variants associated with all four iron biomarkers). Summary-level data for four OA phenotypes, including knee OA, hip OA, total knee replacement, and total hip replacement were obtained from the largest genome-wide meta-analysis with 826,690 individuals. Inverse-variance weighted based on the random-effect model as the main approach was conducted. Weighted median, MR-Egger, and Mendelian randomization pleiotropy residual sum and outlier methods were used as sensitivity MR approaches.

**Results:** Based on liberal instruments, genetically predicted serum iron and transferrin saturation were significantly associated with hip OA and total hip replacement, but not with knee OA and total knee replacement. Statistical evidence of heterogeneity across the MR estimates indicated that mutation rs1800562 was the SNP significantly associated with hip OA in serum iron (odds ratio, OR = 1.48), transferrin saturation (OR = 1.57), ferritin (OR = 2.24), and total-iron binding capacity (OR = 0.79), and hip replacement in serum iron (OR = 1.45), transferrin saturation (OR = 1.25), ferritin (OR = 1.37), and total-iron binding capacity (OR = 0.80).

**Conclusion:** Our study suggests that high iron status might be a causal factor of hip OA and total hip replacement where rs1800562 is the main contributor.

## Introduction

Osteoarthritis (OA) is a common joint disease characterized by loss of articular cartilage, remodeling of the synovitis, and alterations of periarticular structures (Castañeda and Vicente, 2017). Any joint can develop OA, but symptoms linked to OA most commonly affect the knees, hips, hands, and feet (Katz et al., 2021). It is reported that persons with knee or hip OA have excess mortality compared with age-matched controls (Nüesch et al., 2011; Katz et al., 2021). Currently, there is no curative drug for OA. According to the estimation of the World Health Organization, there are about 300 million OA patients worldwide, and the prevalence of OA can reach up to 10%–20% due to the increasing obese and longevous population (GBD, 2017 Disease and Injury Incidence and Prevalence Collaborators, 2018). Given the high health economic burden, a better understanding of the risk factors associated with the occurrence of OA, especially modifiable factors, is needed.

Iron is an essential mineral to various biochemical processes, including DNA synthesis, ATP generation, and oxygen transport, where its absence or depletion can cause abnormal metabolization (Abbaspour et al., 2014). However, excess iron can also be toxic as it would be deposited into organs forming free radicals (Abbaspour et al., 2014). Therefore, disorders of iron homeostasis are involved in a wide scope of diseases (Bogdan et al., 2016). In humans, systemic iron status can be measured by clinical biomarkers: Serum iron, transferrin saturation, ferritin, and total iron-binding capacity (Bell et al., 2021). High serum iron, transferrin saturation, and ferritin signify high iron, while high total iron-binding capacity signifies low iron (Winter et al., 2014). Increasing evidence has found that systemic iron status is associated with OA. For example, high synovial iron was associated with a faster progression of OA in a murine model (Camacho et al., 2016). *In vitro*, iron overload was found to have detrimental effects on various joint components, such as synovium, cartilage, and subchondral bone, leading to synovial hyperplasia and inflammation, abnormal osteoblast function, and chondrocyte apoptosis (van Vulpen et al., 2015). Besides, some epidemiological evidence has shown the relationship between iron and OA risk. In a 2-year longitudinal study with 127 OA patients, radiographic findings showed that higher ferritin was significantly associated with narrower baseline joint space width and higher risks in the prediction of Kellgren-Lawrence grade severity (Kennish et al., 2014). Among 40 subjects with symptomatic knee OA, elevated serum ferritin levels were found to be associated with faster progression of cartilage damage assessed by arthroscopy (Nugzar et al., 2018). However, whether high iron status is a causal factor of OA is unclear because of the inherent defects of observational studies, such as residual confounding and reverse causality.

Mendelian randomization (MR) is a study design that can strengthen the causal inference on exposure-outcome relationships by minimizing the effect of confounding and excluding the potential reverse causality based on the use of genetic variants as instruments (Sheehan et al., 2008). The rationale of the causality assessment in MR is that genetic variants solely associated with the exposure are randomly assorted so that genetic effects on the outcome cannot be affected by potential confounders. Also, genetic variants are not modified by the occurrence or development of any diseases where reverse causality is impossible. For these reasons, MR represents robust indirect evidence of a causal relationship between exposure and outcome if any effect of the selected instruments on diseases is entirely mediated through the exposure (Sheehan et al., 2008).

Therefore, we conducted a 2-sample MR (Lawlor, 2016) study to examine the causal effect of four clinical iron biomarkers on four OA phenotypes, including knee OA, hip OA, total knee replacement, and total hip replacement.

## Materials and methods

**FIGURE 1:**
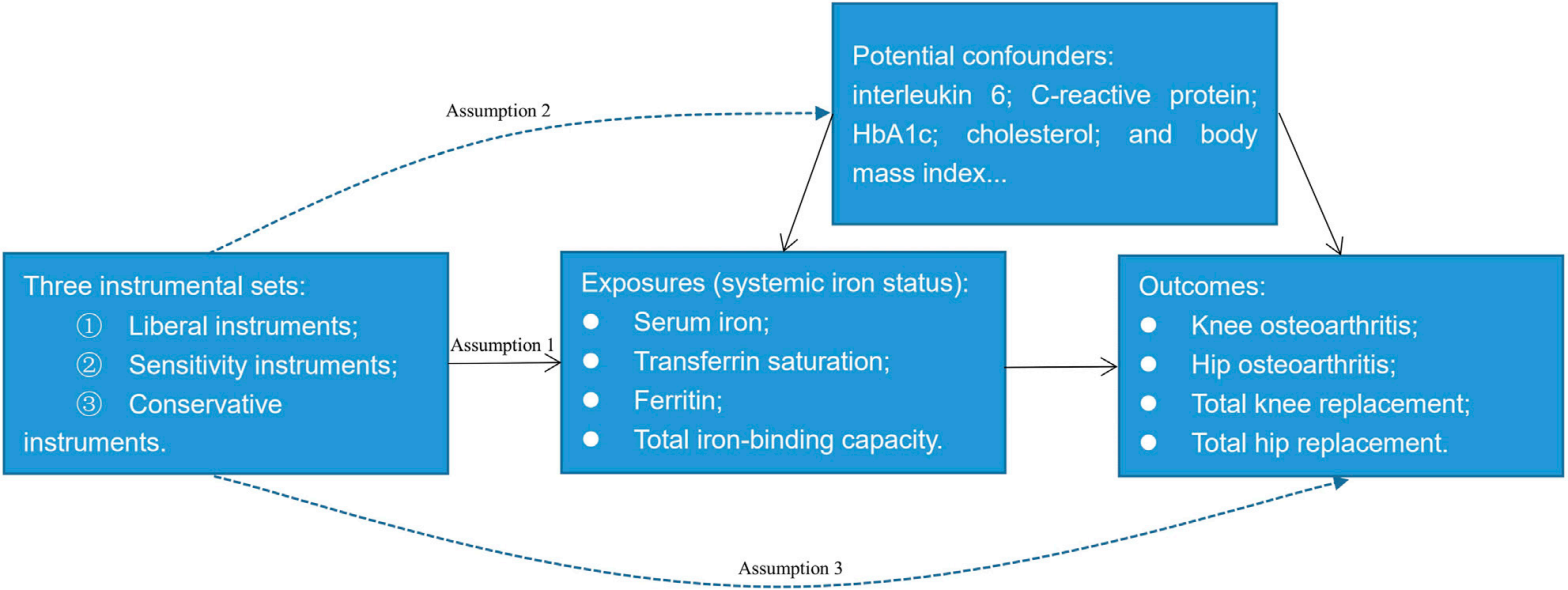
An overview of the Mendelian randomization design. Assumption 1: The instrumental variables must be strongly associated with the exposures; Assumption 2: The instrumental variables must be independent of the potential confounders of the association between the exposure and outcome; Assumption 3: The instrumental variables should not be associated with the outcomes directly.

A 2-sample MR study was conducted to investigate the potential causal relationship between systemic iron status and knee OA, hip OA, total knee replacement, and total hip replacement by using summary-level data. Systemic iron status was comprehensively represented by serum iron, transferrin saturation, ferritin, and total iron-binding capacity. Figure 1 showed the overview of the exposures, the outcomes, and the three assumptions of the genetic instruments for the MR design. Since this study is based on existing publications and public databases, both ethical approval and participant consent have been received by each relevant institutional review committee.

### Genetic instruments for four clinical iron biomarkers

We constructed three sets of genetic instruments for the clinical iron biomarkers: Liberal instruments, sensitivity instruments, and conservative instruments. From the recent meta-analysis of three genome-wide association studies for iron homeostasis biomarkers: serum iron (N = 163,511), transferrin saturation (N = 131,471), ferritin (N = 246,139), and total iron-binding capacity (N = 135,430) (Bell et al., 2021), we selected single nucleotide polymorphisms (SNPs) at the genome-wide significance threshold (*p* < 5 × 10^−8^) and in low linkage disequilibrium (r^2^ < 0.01) as the potential instruments. After excluding five SNPs that are not in our outcome database because their minor allele frequency within the 1000G dataset is 0 (Supplementary Tables S1–S4), 14 SNPs for serum iron, 10 SNPs for transferrin saturation, 37 SNPs for ferritin, and 15 SNPs for total iron-binding capacity were left as the liberal instruments. To avoid the violation of assumption two of the instruments (Figure 1), we searched the Phenoscanner database (http://www.phenoscanner.medschl.cam.ac.uk/) to identify whether these liberal SNPs were associated with potential confounding factors. As interleukin 6, C-reactive protein, glycosylated hemoglobin, cholesterol, and body mass index are potential risk factors of OA (Livshits et al., 2009; Louati et al., 2015; Zheng and Chen, 2015; Farnaghi et al., 2017; Kozijn et al., 2019), we further excluded SNPs that were strongly associated with these phenotypes (*p* < 5 × 10^−8^) leaving 12 SNPs for serum iron, nine SNPs for transferrin saturation, 27 SNPs for ferritin, and 14 SNPs for total iron-binding capacity as the sensitivity instruments (Supplementary Tables S1–S4). We did not exclude rs855791 from *TMPRSS6*, rs1799945 from *HFE*, and rs1800562 from *HFE* here since they were missense mutations of TMPRSS6 or HFE. While HFE and TMPRSS6 involve the signaling cascade of iron homeostasis directly (Bell et al., 2021), these three SNPs may have a vertical effect from iron to the potential confounding factors, namely glycosylated hemoglobin, low-density lipoprotein, and total cholesterol mentioned in the Supplementary Tables. Thus, these three SNPs may have vertical pleiotropy with the possible confounding factors which does not violate assumption two of MR (Davies et al., 2018) (Figure 1). Similar instrument selection criteria are applied to evade excluding vertical SNPs (Chong et al., 2022). Conservative instruments were constructed where only SNPs associated with all four clinical iron biomarkers were eligible; therefore, rs1800562, rs1799945, rs855791, and rs57659670 were used as conservative instruments. Because the MR estimates were for each iron biomarker, we set the liberal instruments that have greater power as the main instrument. The strength of the instruments was evaluated by the F statistic using the formula F = R^2^(N-2)/(1-R^2^) (Palmer et al., 2012), where R^2^ is the proportion of the variance of the four clinical iron biomarkers explained by the genetic variant and N is the sample size of the gene-each of the iron biomarker association. Thus, the computed F statistics range from 5136 to 6388 for serum iron, from 7012 to 8489 for transferrin saturation, from 1644 to 5844 for ferritin, and from 4206 to 9724 for total iron-binding capacity which was much greater than 10 demonstrating sufficient statistical strength (Burgess et al., 2013).

### Genetic associations with four OA phenotypes

To obtain the associations of the genetic instruments with OA, summary-level data were extracted from the largest genome-wide meta-analysis to date across 826,690 individuals with 177,517 cases and 649,173 controls. The mean age (standard deviation) is 62.4 (11.9) for the cases and 52.4 (17.4) for the controls. The percentage of females is 62% for cases and 52% for controls. The meta-analysis was a combination of data from 13 cohorts with a population of >97% European descent (Boer et al., 2021).

In this study, data from knee OA (n = 62,497), hip OA (n = 36,445), total knee replacement (n = 18,200), total hip replacement (n = 23,021), and a max of healthy controls (n = 333,557) were used (Boer et al., 2021). Knee (or hip) OA was defined as OA or joint replacement at the knee joint (or hip joint). Total knee (or hip) replacement was defined as having undergone total knee (or hip) replacement due to OA in the knee joint (or hip joint). All the definitions of the cases were self-reported, clinically diagnosed, ICD10, codes, or radiographic depending on the data available in the cohort. Controls were osteoarthritis-free or population-based with or without ICD code exclusions. Detailed association estimates for the genetic instruments with the four OA phenotypes were provided in Supplementary Tables S1–S4.

### MR estimates

To derive MR estimates, the inverse-variance weighted method (IVW) was used as the main approach. This method first assesses the effect of each SNP on the outcome by calculating the Wald ratio and then uses the inverse variance of SNPs as weights to obtain a combined causal effect (Lee et al., 2016). Random-effects model was selected for this method because the fixed-effects models may be at risk of yielding artificially precise estimates in the presence of heterogeneity (Burgess et al., 2017). Furthermore, heterogeneity across the instrumental SNPs was evaluated using I^2^ statistics and Cochran’s Q test (Higgins et al., 2003). For each SNP, the MR estimates were obtained from the Wald ratio method with standard error derived using the Delta method (Thompson et al., 2016). The MR estimates are presented using odds ratios which are scaled to one standard deviation increment of genetically predicted clinical iron biomarkers.

To investigate the robustness of the findings and assess the possible horizontal pleiotropy, we further performed the weighted median, MR-Egger, and Mendelian randomization pleiotropy residual sum and outlier (MR-PRESSO) methods as sensitivity MR approaches. The weighted median method first orders the ratio estimate of each instrument SNP on the outcome by its magnitude of weight and then produces an overall MR estimate based on the median value with standard error derived using the parametric bootstrap method (Bowden et al., 2016). The MR-Egger method can detect the overall horizontal pleiotropy of genetic instruments by examining whether the intercept of the association between exposure and outcome differs from zero (Burgess and Thompson, 2017). However, the estimates of MR-Egger are generally small especially when the number of instruments is small (Burgess and Thompson, 2017), and thus, we did not use MR-Egger when using conservative instruments. MR-PRESSO identifies possible pleiotropic outliers and generates estimates after the outliers are removed (Verbanck et al., 2018). Moreover, a leave-one-out analysis was computed where we excluded one SNP at a time and conducted IVW on the rest SNPs to test whether any single variant was driving the causal association between exposure and outcome (Burgess et al., 2017).

Because four OA outcomes and four biomarkers were involved in the analyses, we set a Bonferroni corrected *p*-value of 0.003 (0.05/16) as the statistical significance level. All statistical data analyses were conducted with R software. The MendelianRandomization and the forestplot packages were used to facilitate the MR analyses and display the results (Yavorska and Burgess, 2017).

### Repeated analysis in the second-largest meta-analysis of OA GWAS

We also repeated our analyses in the second-largest meta-analysis of OA GWAS (Tachmazidou et al., 2019). This meta-analysis was a combination of data from UK Biobank and Arthritis Research UK Osteoarthritis Genetics (arcOGEN) with 455,221 individuals of European descent only (Tachmazidou et al., 2019). Due to the large number of OA participants in these two cohorts, there are some overlapping participants between the largest and the second-largest meta-analysis of OA GWAS. As the summary data of total knee replacement and total hip replacement were not available in this meta-analysis, we only repeated our analyses in the knee OA (n = 24,955) and hip OA (n = 15,704) with healthy controls (n = 378,169) (Tachmazidou et al., 2019). The OA definitions were from self-report and hospital records in UK Biobank and were from total joint replacement records or radiographic evidence of disease in arcOGEN.

## Results

**FIGURE 2:**
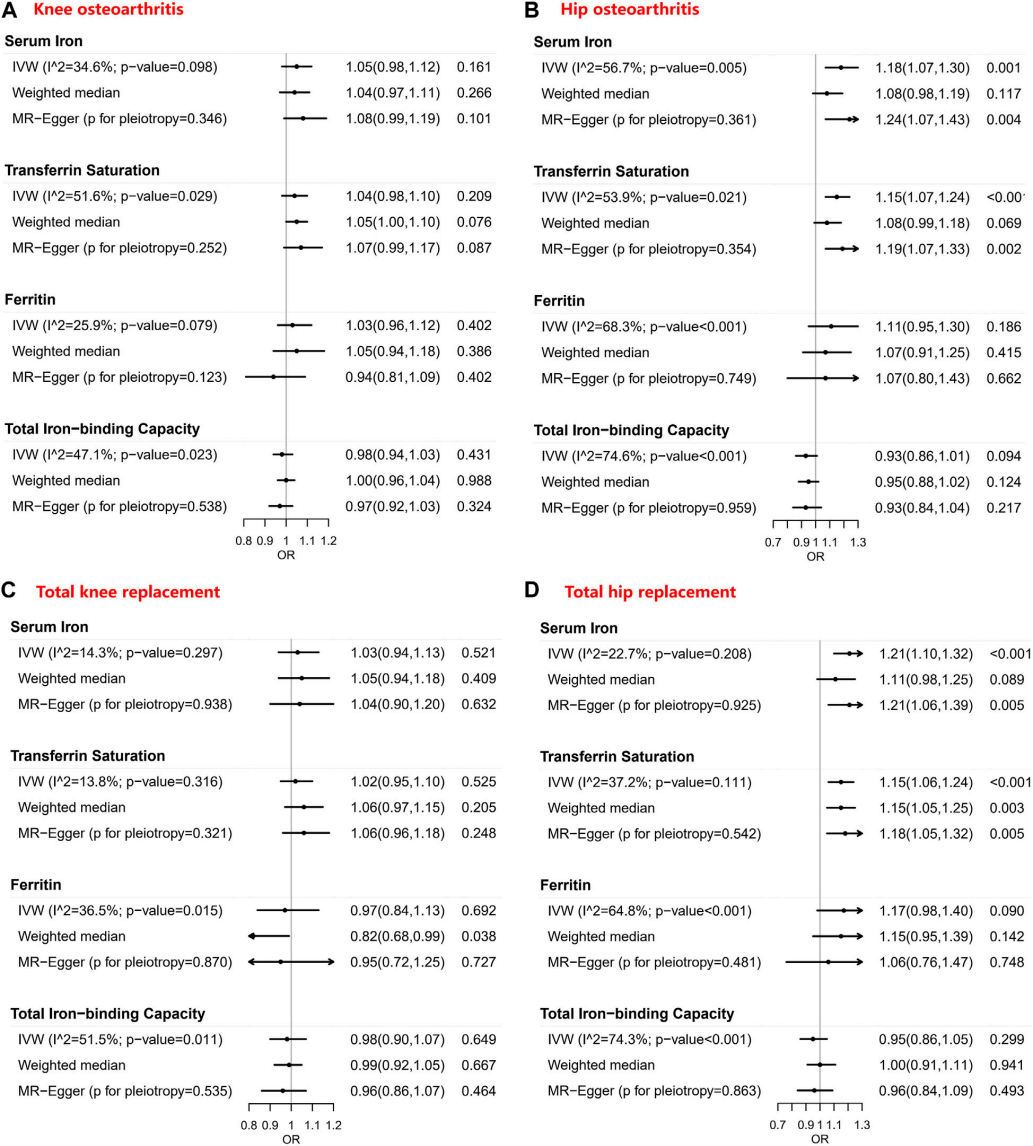
Forest plots of Mendelian randomization estimates for the association between genetically predicted systemic iron biomarkers with liberal instruments and knee osteoarthritis, hip osteoarthritis, total knee replacement, and total hip replacement. OR: Odds ratio; CI: Compatibility/confidence interval; IVW: Inverse-variance weighted. Points indicate the odds ratio per standard deviation increment increase in the iron biomarkers. Error bars indicate 95% compatibility/confidence intervals. **(A)** for knee osteoarthritis, **(B)** for hip osteoarthritis, **(C)** for total knee replacement, and **(D)** for total hip replacement.

Tables 1–4 show the associations of the four systemic iron status biomarkers and knee OA, hip OA, knee replacement, and hip replacement using the three instrument sets. In the IVW analyses using liberal instruments, genetically predicted serum iron and transferrin saturation were found to be significantly associated with hip OA and hip replacement, and the other associations were not evident (Tables 1–4; Figure 2). The odds ratios of hip OA were 1.18 (95% CI: 1.07–1.30) per one standard deviation increment in serum iron and 1.15 (95% CI: 1.07–1.24) in transferrin saturation (Tables 1–4; Figure 2).

**TABLE 1:** Associations of the instrumental SNPs with four systemic iron status biomarkers and knee osteoarthritis.

Iron biomarkers	Analysis method	Liberal instruments			Sensitivity instruments			Conservative instruments		
		OR (95% CI)	*p*-value*	Heterogeneity: I^2^; *p*-value	OR (95% CI)	*p*-value*	Heterogeneity: I^2^; *p*-value	OR (95% CI)	*p*-value*	Heterogeneity: I^2^; *p*-value
Serum iron	IVW	1.05 (0.98, 1.12)	0.161	34.6%; 0.098	1.06 (1.00, 1.12)	0.040	14.8%; 0.300	1.06 (0.95, 1.17)	0.298	70.4%; 0.017
	Weighted median	1.04 (0.97, 1.11)	0.266		1.04 (0.97, 1.11)	0.270		1.04 (0.97, 1.11)	0.265	
	MR-Egger	1.08 (0.99, 1.19)	0.101		1.07 (0.98, 1.17)	0.114		-	-	
	MR-PRESSO	1.05 (0.98, 1.12)	0.184		1.06 (0.99, 1.13)	0.060		-	-	
Transferrin saturation	IVW	1.04 (0.98, 1.10)	0.209	51.6%; 0.029	1.05 (0.99, 1.10)	0.079	35.5%; 0.134	1.05 (0.97, 1.13)	0.226	67.7%; 0.026
	Weighted median	1.05 (0.99, 1.10)	0.076		1.05 (0.99, 1.10)	0.064		1.05 (1.00, 1.10)	0.049	
	MR-Egger	1.07 (0.99, 1.17)	0.087		1.07 (0.99, 1.15)	0.084		-	-	
	MR-PRESSO	1.04 (0.98, 1.10)	0.241		1.05 (0.99, 1.11)	0.117		-	-	
Ferritin	IVW	1.03 (0.96, 1.12)	0.402	25.9%; 0.079	1.00 (0.91, 1.10)	0.958	27.1%; 0.110	1.01 (0.78, 1.31)	0.953	78.3%; 0.003
	Weighted median	1.05 (0.94, 1.18)	0.386		1.04 (0.91, 1.18)	0.606		1.12 (0.91, 1.38)	0.272	
	MR-Egger	0.94 (0.81, 1.09)	0.402		0.96 (0.80, 1.13)	0.601		-	-	
	MR-PRESSO	1.04 (1.00, 1.07)	0.034		1.00 (0.92, 1.09)	0.958		-	-	
Total iron-binding capacity	IVW	0.98 (0.94, 1.03)	0.431	47.1%; 0.023	0.98 (0.94, 1.02)	0.340	39.5%; 0.064	0.94 (0.85, 1.05)	0.267	69.4%; 0.020
	Weighted median	1.00 (0.96, 1.04)	0.988		1.00 (0.96, 1.04)	0.987		0.93 (0.88, 0.99)	0.018	
	MR-Egger	0.97 (0.92, 1.03)	0.324		0.98 (0.92, 1.03)	0.436		-	-	
	MR-PRESSO	0.98 (0.94, 1.03)	0.444		0.98 (0.94, 1.02)	0.357		-	-	

*Bolded *p* values indicate a value of <0.003.

IVW, inverse variance weighted; MR-PRESSO, mendelian randomization pleiotropy residual sum and outlier; OR, odds ratio; CI, compatibility/confidence interval.

**TABLE 2:** Associations of the instrumental SNPs with four systemic iron status biomarkers and hip osteoarthritis.

Iron biomarkers	Analysis method	Liberal instruments			Sensitivity instruments			Conservative instruments		
		OR (95% CI)	*p*-value*	Heterogeneity: I^2^; *p*-value	OR (95% CI)	*p*-value*	Heterogeneity: I^2^; *p*-value	OR (95% CI)	*p*-value*	Heterogeneity: I^2^; *p*-value
Serum iron	IVW	1.18 (1.07, 1.30)	**0.001**	56.7%; 0.005	1.18 (1.06, 1.31)	**0.003**	62.7%; 0.002	1.18 (0.97, 1.43)	0.100	86.5%; <0.001
	Weighted median	1.08 (0.98, 1.19)	0.117		1.08 (0.98, 1.19)	0.126		1.08 (0.98, 1.19)	0.119	
	MR-Egger	1.24 (1.07, 1.43)	0.004		1.25 (1.07, 1.47)	0.006		-	-	
	MR-PRESSO	1.09 (0.99, 1.19)	0.068^#^		1.08 (0.98, 1.20)	0.113^#^		-	-	
Transferrin saturation	IVW	1.15 (1.07, 1.24)	**<0.001**	53.9%; 0.021	1.15 (1.06, 1.25)	**0.001**	58.7%; 0.013	1.16 (1.02, 1.31)	0.022	80.5%; 0.002
	Weighted median	1.08 (0.99, 1.18)	0.069		1.15 (1.05, 1.25)	**0.002**		1.14 (1.07, 1.22)	**<0.001**	
	MR-Egger	1.09 (1.07, 1.33)	**0.002**		1.09 (1.06, 1.34)	**0.003**		-	-	
	MR-PRESSO	1.15 (1.04, 1.27)	0.006		1.15 (1.04, 1.28)	0.009		-	-	
Ferritin	IVW	1.11 (0.95, 1.30)	0.186	68.3%; <0.001	1.08 (0.90, 1.31)	0.400	72.6%; <0.001	1.25 (0.75, 2.10)	0.386	91.1%; <0.001
	Weighted median	1.07 (0.91, 1.25)	0.415		1.00 (0.84, 1.20)	0.977		1.26 (0.94, 1.70)	0.117	
	MR-Egger	1.07 (0.80, 1.43)	0.662		1.08 (0.75, 1.54)	0.689		-	-	
	MR-PRESSO	1.10 (0.97, 1.26)	0.145^#^		1.02 (0.86, 1.21)	0.835^#^		-	-	
Total iron-binding capacity	IVW	0.93 (0.86, 1.01)	0.094	74.6%; <0.001	0.93 (0.86, 1.02)	0.109	76.4%; <0.001	0.82 (0.71, 0.93)	**0.003**	72.3%; 0.013
	Weighted median	0.95 (0.88, 1.02)	0.124		0.95 (0.88, 1.02)	0.136		0.80 (0.75, 0.87)	**<0.001**	
	MR-Egger	0.93 (0.84, 1.04)	0.217		0.93 (0.83, 1.04)	0.234		-	-	
	MR-PRESSO	0.93 (1.02, 0.86)	0.116		0.93 (0.85, 1.02)	0.133		-	-	

*Bolded *p* values indicate a value of <0.003.

#Presented with outlier-corrected value because the global test of the pleiotropy is significant (*p* < 0.05).

IVW, inverse variance weighted; MR-PRESSO, mendelian randomization pleiotropy residual sum and outlier; OR, odds ratio; CI, compatibility/confidence interval.

**TABLE 3:** Associations of the instrumental SNPs with four systemic iron status biomarkers and total knee replacement.

Iron biomarkers	Analysis method	Liberal instruments			Sensitivity instruments			Conservative instruments		
		OR (95% CI)	*p*-value*	Heterogeneity: I^2^; *p*-value	OR (95% CI)	*p*-value*	Heterogeneity: I^2^; *p*-value	OR (95% CI)	*p*-value*	Heterogeneity: I^2^; *p*-value
Serum iron	IVW	1.03 (0.94, 1.13)	0.521	14.3%; 0.297	1.05 (0.96, 1.15)	0.304	5.5%; 0.392	1.04 (0.90, 1.20)	0.609	56.9%; 0.073
	Weighted median	1.05 (0.94, 1.18)	0.409		1.06 (0.94, 1.19)	0.358		1.06 (0.94, 1.19)	0.334	
	MR-Egger	1.04 (0.87, 1.20)	0.632		1.02 (0.89, 1.18)	0.754		-	-	
	MR-PRESSO	1.03 (0.94, 1.13)	0.532		1.05 (0.95, 1.15)	0.326		-	-	
Transferrin saturation	IVW	1.02 (0.95, 1.10)	0.525	13.8%; 0.316	1.04 (0.97, 1.11)	0.330	0%; 0.508	1.04 (0.93, 1.16)	0.539	55.4%; 0.081
	Weighted median	1.06 (0.97, 1.15)	0.205		1.06 (0.97, 1.15)	0.194		1.06 (0.98, 1.16)	0.160	
	MR-Egger	1.06 (0.96, 1.18)	0.248		1.06 (0.96, 1.17)	0.275		-	-	
	MR-PRESSO	1.02 (0.95, 1.10)	0.540		1.04 (0.97, 1.11)	0.337		-	-	
Ferritin	IVW	0.97 (0.84, 1.13)	0.692	36.5%; 0.015	0.94 (0.79, 1.12)	0.514	41.0%; 0.020	0.95 (0.69, 1.31)	0.766	59.2%; 0.061
	Weighted median	0.82 (0.68, 0.99)	0.038		0.82 (0.66, 1.00)	0.053		0.90 (0.67, 1.21)	0.501	
	MR-Egger	0.95 (0.72, 1.25)	0.727		0.91 (0.66, 1.26)	0.567		-	-	
	MR-PRESSO	0.97 (0.84, 1.12)	0.695		0.94 (0.79, 1.12)	0.521		-	-	
Total iron-binding capacity	IVW	0.98 (0.90, 1.07)	0.649	51.5%; 0.011	0.98 (0.90, 1.06)	0.587	50.0%; 0.017	0.96 (0.83, 1.10)	0.551	55.7%; 0.080
	Weighted median	0.99 (0.92, 1.05)	0.667		0.99 (0.92, 1.05)	0.669		0.95 (0.86, 1.04)	0.269	
	MR-Egger	0.96 (0.86, 1.07)	0.464		0.97 (0.87, 1.08)	0.564		-	-	
	MR-PRESSO	0.98 (0.90, 1.06)	0.656		0.98 (0.91, 1.06)	0.596		-	-	

*Bolded *p* values indicate a value of <0.003.

IVW, inverse variance weighted; MR-PRESSO, mendelian randomization pleiotropy residual sum and outlier; OR, odds ratio; CI, compatibility/confidence interval.

**TABLE 4:** Associations of the instrumental SNPs with four systemic iron status biomarkers and total hip replacement.

Iron biomarkers	Analysis method	Liberal instruments			Sensitivity instruments			Conservative instruments		
		OR (95% CI)	*p*-value*	Heterogeneity: I^2^; *p*-value	OR (95% CI)	*p*-value*	Heterogeneity: I^2^; *p*-value	OR (95% CI)	*p*-value*	Heterogeneity: I^2^; *p*-value
Serum iron	IVW	1.21 (1.10, 1.32)	**<0.001**	22.7%; 0.208	1.20 (1.09, 1.32)	**<0.001**	27.8%; 0.172	1.19 (1.01, 1.41)	0.041	73.1%; 0.011
	Weighted median	1.11 (0.98, 1.25)	0.089		1.10 (0.98, 1.24)	0.116		1.10 (0.98, 1.24)	0.116	
	MR-Egger	1.21 (1.06, 1.29)	0.005		1.23 (1.07, 1.42)	0.004		-	-	
	MR-PRESSO	1.21 (1.08, 1.35)	**0.001**		1.20 (1.07, 1.36)	**0.003**		-	-	
Transferrin saturation	IVW	1.15 (1.06, 1.24)	**<0.001**	37.2%; 0.111	1.15 (1.06, 1.25)	**0.001**	44.2%; 0.073	1.16 (1.06, 1.28)	**0.002**	53.7%; 0.091
	Weighted median	1.15 (1.05, 1.25)	**0.003**		1.13 (1.03, 1.24)	0.007		1.14 (1.06, 1.24)	**0.001**	
	MR-Egger	1.18 (1.05, 1.32)	0.005		1.18 (1.04, 1.33)	0.009		-	-	
	MR-PRESSO	1.15 (1.04, 1.27)	0.007		1.15 (1.03, 1.28)	0.011		-	-	
Ferritin	IVW	1.17 (0.98, 1.40)	0.090	64.8%; <0.001	1.13 (0.91, 1.40)	0.277	69.5%; <0.001	1.34 (0.86, 2.08)	0.201	82.6%; <0.001
	Weighted median	1.15 (0.95, 1.39)	0.142		0.93 (0.75, 1.14)	0.481		1.33 (0.97, 1.81)	0.074	
	MR-Egger	1.06 (0.76, 1.47)	0.748		1.08 (0.72, 1.63)	0.702		-	-	
	MR-PRESSO	1.10 (0.93, 1.29)	0.284^#^		1.07 (0.87, 1.30)	0.536		-	-	
Total iron-binding capacity	IVW	0.95 (0.86, 1.05)	0.299	74.3%; <0.001	0.95 (0.85, 1.05)	0.326	76.0%; <0.001	0.81 (0.74, 0.89)	**<0.001**	15.1%; 0.317
	Weighted median	1.00 (0.91,1.11)	0.941		1.00 (0.91, 1.10)	0.932		0.81 (0.74, 0.88)	**<0.001**	
	MR-Egger	0.96 (0.84, 1.09)	0.493		0.95 (0.83, 1.09)	0.493		-	-	
	MR-PRESSO	0.95 (0.85, 1.05)	0.316		0.95 (0.85, 1.06)	0.343		-	-	

*Bolded *p* values indicate a value of <0.003.

#Presented with outlier-corrected value because the global test of the pleiotropy is significant (*p* < 0.05).

IVW, inverse variance weighted; MR-PRESSO, mendelian randomization pleiotropy residual sum and outlier; OR, odds ratio; CI, compatibility/confidence interval.

Similar results were seen in analyses using sensitivity instruments. In the analyses using conservative instruments, genetically predicted total iron-binding capacity was negatively associated with hip OA and hip replacement with ORs ranging from 0.80 to 0.82 (Tables 2, 4). However, statistical evidence of heterogeneity across the MR estimates was found especially for hip OA and hip replacement (Tables 2, 4; Figure 2). For example, in the MR estimates of ferritin on hip OA and hip replacement, the I^2^ of the heterogeneity was 64.8%–91.1%. When we used the Wald ratio method to assess the MR estimates of four individual SNPs (rs57659670, rs855791, rs1799945, and rs1800562) with hip OA and hip replacement, rs1800562 was the SNP that was significantly associated with hip OA in serum iron (OR = 1.48; 95% CI: 1.29-1.68), transferrin saturation (OR = 1.57; 95% CI: 1.17-1.37), ferritin (OR = 2.24; 95% CI: 1.71-2.94), and total-iron binding capacity (OR = 0.79; 95% CI: 0.73-0.86), and hip replacement in serum iron (OR = 1.45; 95% CI: 1.25-1.69), transferrin saturation (OR = 1.25; 95% CI: 1.14-1.37), ferritin (OR = 1.37; 95% CI: 1.58-2.99), and total-iron binding capacity (OR = 0.80; 95% CI: 0.73-0.88). The other three SNPs did not show evident associations with hip OA or hip replacement (data not shown). Also, the leave-one-out analyses demonstrated that the causal effect of systemic iron status on hip OA and total hip replacement was mainly resulted from rs1800562 (Supplementary Table S5). We repeated the MR estimates of the four systemic iron status biomarkers on knee OA and hip OA in the second-largest meta-analysis of OA GWAS, and similar results were shown in Supplementary Table S6. That is, serum iron, transferrin saturation, and total iron-binding capacity were associated with hip OA, and the other associations were not evident.

## Discussion

The present 2-sample MR study used the largest public dataset to date to comprehensively evaluate the causal role of systemic iron status for knee OA, hip OA, total knee replacement, and total hip replacement. In a pattern concordant with an effect on systemic iron status, we found that genetically predicted clinical iron biomarkers were significantly associated with hip OA and total hip replacement. Given the heterogeneity across the genetic instrumental variables, these associations were mainly resulted from rs1800562, a missense mutation of the *HFE* gene.

Our findings are in line with previous conclusions from observational and experimental studies that iron is involved in the development of OA. In an observational study, synovial fluid iron concentrations determined by the colorimetric method were significantly higher in OA patients compared to the controls (Yazar et al., 2005). Another 2-year follow-up study reported that higher iron status was associated with more severe radiographic progression in knee OA patients (Kennish et al., 2014). In murine models, cellular iron accumulation in the knee joint resulted from systemic iron overload could induce the early onset of OA and accelerate OA progression *via* compromising chondrocyte metabolism and over-expressing local inflammatory mediators (Camacho et al., 2016; Simão et al., 2019; Burton et al., 2020). Several studies using the MR method also assessed the association between iron and OA, though the results were inconsistent. Using *HFE* genotype (rs1800562 & rs1799945) instrumented transferrin saturation as the iron load, Pilling et al. (2019) found that homozygotes of rs1800562 or compound heterozygotes of rs1800562 & rs1799945 were positively associated with incident OA in both men and women. Using rs800562, rs1799945, and rs855791 as the instruments of serum iron, no significant association was observed between iron levels and overall OA in UK Biobank (Zhou et al., 2020; Zhou et al., 2021), but per standard deviation increment in iron was associated with increased risk of OA in males (Zhou et al., 2021). Using rs800562, rs1799945, rs855791, and rs8177240 as the instruments of nutritional iron and summary data from the UK Biobank and arcOGEN cohorts, no significant association was observed between the iron and knee OA, hip OA, or overall OA, but the positive causal effect of iron levels on overall OA was observed in females (Qu et al., 2020). Using the instruments from the Genetics of Iron Status Consortium and the summary-level data of outcomes from the UK Biobank and arcOGEN cohorts, Xu et al. (2022) found that transferrin saturation was positively associated with both knee OA and hip OA, transferrin was negatively associated with hip OA, but serum iron and ferritin did not show a prominent effect on OA outcomes. In our study, we assessed the iron status using four biomarkers with three instrument sets while the previous studies used one biomarker with three or four instruments. Our study used the largest meta-analysis data for OA while the others included the relatively smaller size of OA patients. The inconsistencies between the previous studies with the present study can be partly explained by the different biomarkers with non-specific instruments and underpowered samples. Thus, the conclusion from our study may be more representative of the association between iron status and OA.

Our findings only suggest causal associations among hip OA and total hip replacement, but not knee OA or total knee replacement. This could be explained by the fact that genetic heritability contributes more to hip OA than knee OA [14.7% for knee OA and 51.9% for hip OA (Tachmazidou et al., 2019)], and the exposures in the MR study are genetically predicted. In the main analyses and the repeated analyses, we only found causal associations between hip OA & total hip replacement and serum iron, transferrin saturation, & total iron-binding capacity, but not ferritin. This can be partly explained by the heterogeneity among the variants in the analyses (I^2^ = 91% in hip OA and I^2^ = 83% in the total hip replacement). Another possible explanation for this is that ferritin is a representative biomarker of body iron stores in a non-inflammatory state (Adams, 2015; Bell et al., 2021), while OA is regarded as an inflammatory disease (Dainese et al., 2022), and thus, there might be a weak relationship between ferritin and OA. A potential concern for MR conclusions relates to the horizontal pleiotropy of the instruments. Our MR study does not have a such concern, because the findings were robust to analyses using sensitivity instruments that exclude variants associated with potential confounders. Though rs1800562 from the *HFE* gene is associated with potential confounders glycosylated hemoglobin, low-density lipoprotein, and total cholesterol, we did not exclude this variant in our analyses. This is because the HFE protein encoded by the *HFE* gene is the central regulator of systemic iron homeostasis and has no other well-established roles (Barton et al., 2015), any effect of the second phenotype may probably be acting downstream of iron status, rather than independent of it. Therefore, rs1800562 may have a vertical pleiotropy with the outcome which does not violate the assumption of MR (Davies et al., 2018). However, there is still a possibility that rs1800562 has other horizontal effects on the outcomes independent of iron.

Besides the causal evidence of the MR relationships, the biological plausibility adds further support for the possibility of causality. Hereditary hemochromatosis is a common disease in Europeans characterized by iron overload in multiple organs where the mutation of rs1800562 is the main contributor (Husar-Memmer et al., 2014). It is reported that people with hemochromatosis are at increased risk of OA and OA is one of the most frequent complications in people with hemochromatosis (Elmberg et al., 2013; Husar-Memmer et al., 2014). The missense mutation rs1800562 from the *HFE* gene affects the synthesis of HFE protein which is associated with the regulation of the circulating iron by regulating the interaction of the transferrin receptor with transferrin. Studies showed that excess iron could act as a catalyst to produce a large number of reactive oxygen species which are deleterious agents involved in OA cartilage degradation (Henrotin et al., 2003; Yang et al., 2018). Also, evidence showed that iron homeostasis dysregulation could lead to the secretion of pro-inflammatory cytokines which promote chondrocytes apoptosis and subsequent OA cartilage degeneration (Simão et al., 2019; Jing et al., 2021).

Since high iron status is a treatable condition, our findings have important clinical and public health implications. It is reported that phlebotomy, oral chelation, and dietary changes are options for people with iron overload (Palmer et al., 2018). There may be a benefit to the careful reduction of iron indices for reducing OA risk in people with high iron status, especially among hemochromatosis patients or people with the mutation rs1800562. However, the interpretation of the MR finding that iron decrement might be a benefit for OA patients requires justification, because MRs are the effects of lifelong exposures while interventions consider short-term pharmacological treatments. Taken together, iron decrement could be an attractive new target for hip OA treatment which needs to be validated in randomized controlled trials in the future.

The major strength of the present study is the 2-sample MR design, which minimizes the confounding and reverse causality seen in observational studies. We comprehensively investigated the causation of the systemic iron status in four representative biomarkers to knee OA, hip OA, total knee replacement, and total hip replacement using the largest summary-level data. Also, we used three instrument sets to obtain robust conclusions. Furthermore, we repeated our analyses in the second largest OA GWAS dataset with European descent, thereby diminishing bias from population stratification. However, we must acknowledge some potential limitations. First, rs1800562 was the only SNP associated with OA which needs to be verified in another dataset independent from the UK Biobank study and the arcOGEN consortium. Second, the findings of our study relate to patterns of iron status largely within the normal range, and thus, cannot be used to make inferences on the effect of abnormally high or low serum iron levels. Third, our study does not offer insight into whether the estimates are equally applicable to both men and women. Despite these limitations, the results of this work show consistent and biologically plausible effects.

In summary, the present MR findings suggest that high iron status might be a causal factor of hip OA and total hip replacement where rs1800562 is the main contributor. Given the modifiable nature of the iron status, further clinical trials are warranted to validate the therapeutic role of iron decrement in people with hip OA, especially among those with the mutation rs1800562.

## Data Availability

The datasets presented in this study can be found in online repositories. The names of the repository/repositories and accession number(s) can be found below: https://github.com/yzhang666666/IronMR_23022023.

## References

[B1] Abbaspour N. Hurrell R. Kelishadi R. (2014). Review on iron and its importance for human health. J. Res. Med. Sci. 19, 164–174. 24778671 PMC3999603

[B2] Adams P. C. (2015). Epidemiology and diagnostic testing for hemochromatosis and iron overload. Int. J. Lab. Hematol. 37 (1), 25–30. 10.1111/ijlh.12347 25976957

[B3] Barton J. C. Edwards C. Q. Acton R. T. (2015). HFE gene: Structure, function, mutations, and associated iron abnormalities. Gene 574, 179–192. 10.1016/j.gene.2015.10.009 26456104 PMC6660136

[B4] Bell S. Rigas A. S. Magnusson M. K. Ferkingstad E. Allara E. Bjornsdottir G. (2021). A genome-wide meta-analysis yields 46 new loci associating with biomarkers of iron homeostasis. Commun. Biol. 4, 156. 10.1038/s42003-020-01575-z 33536631 PMC7859200

[B5] Boer C. G. Hatzikotoulas K. Southam L. Stefansdottir L. Zhang Y. Coutinho de Almeida R. (2021). Deciphering osteoarthritis genetics across 826,690 individuals from 9 populations. Cell. 184, 6003–6005. 10.1016/j.cell.2021.11.003 34822786 PMC8658458

[B6] Bogdan A. R. Miyazawa M. Hashimoto K. Tsuji Y. (2016). Regulators of iron homeostasis: New players in metabolism, cell death, and disease. Trends Biochem. Sci. 41, 274–286. 10.1016/j.tibs.2015.11.012 26725301 PMC4783254

[B7] Bowden J. Davey Smith G. Haycock P. C. Burgess S. (2016). Consistent estimation in mendelian randomization with some invalid instruments using a weighted median estimator. Genet. Epidemiol. 40, 304–314. 10.1002/gepi.21965 27061298 PMC4849733

[B8] Burgess S. Bowden J. Fall T. Ingelsson E. Thompson S. G. (2017). Sensitivity analyses for robust causal inference from mendelian randomization analyses with multiple genetic variants. Epidemiol. Camb. Mass) 28, 30–42. 10.1097/ede.0000000000000559 PMC5133381 27749700

[B9] Burgess S. Butterworth A. Thompson S. G. (2013). Mendelian randomization analysis with multiple genetic variants using summarized data. Genet. Epidemiol. 37, 658–665. 10.1002/gepi.21758 24114802 PMC4377079

[B10] Burgess S. Thompson S. G. (2017). Interpreting findings from Mendelian randomization using the MR-Egger method. Eur. J. Epidemiol. 32, 377–389. 10.1007/s10654-017-0255-x 28527048 PMC5506233

[B11] Burton L. H. Radakovich L. B. Marolf A. J. Santangelo K. S. (2020). Systemic iron overload exacerbates osteoarthritis in the strain 13 Guinea pig. Osteoarthr. Cartil. 28, 1265–1275. 10.1016/j.joca.2020.06.005 PMC7484276 32629162

[B12] Camacho A. Simão M. Ea H. K. Cohen-Solal M. Richette P. Branco J. (2016). Iron overload in a murine model of hereditary hemochromatosis is associated with accelerated progression of osteoarthritis under mechanical stress. Osteoarthr. Cartil. 24, 494–502. 10.1016/j.joca.2015.09.007 26403062

[B13] Castañeda S. Vicente E. F. (2017). Osteoarthritis: More than cartilage degeneration. Clin. Rev. Bone Mineral Metabolism 15, 69–81. 10.1007/s12018-017-9228-6

[B14] Chong M. Mohammadi-Shemirani P. Perrot N. Nelson W. Morton R. Narula S. (2022). GWAS and ExWAS of blood mitochondrial DNA copy number identifies 71 loci and highlights a potential causal role in dementia. eLife 11, e70382. 10.7554/eLife.70382 35023831 PMC8865845

[B15] Dainese P. Wyngaert K. V. De Mits S. Wittoek R. Van Ginckel A. Calders P. (2022). Association between knee inflammation and knee pain in patients with knee osteoarthritis: A systematic review. Osteoarthr. Cartil. 30, 516–534. 10.1016/j.joca.2021.12.003 34968719

[B16] Davies N. M. Holmes M. V. Davey Smith G. (2018). Reading mendelian randomisation studies: A guide, glossary, and checklist for clinicians. BMJ 362, k601. 10.1136/bmj.k601 30002074 PMC6041728

[B17] Elmberg M. Hultcrantz R. Simard J. F. Carlsson A. Askling J. (2013). Increased risk of arthropathies and joint replacement surgery in patients with genetic hemochromatosis: A study of 3,531 patients and their 11,794 first-degree relatives. Arthritis Care Res. Hob. 65, 678–685. 10.1002/acr.21883 23139229

[B18] Farnaghi S. Crawford R. Xiao Y. Prasadam I. (2017). Cholesterol metabolism in pathogenesis of osteoarthritis disease. Int. J. Rheum. Dis. 20, 131–140. 10.1111/1756-185x.13061 28378420

[B19] GBD 2017 Disease and Injury Incidence and Prevalence Collaborators (2018). Global, regional, and national incidence, prevalence, and years lived with disability for 354 diseases and injuries for 195 countries and territories, 1990-2017: A systematic analysis for the global burden of disease study 2017. Lancet 392, 1789–1858. 10.1016/s0140-6736(18)32279-7 30496104 PMC6227754

[B20] Henrotin Y. E. Bruckner P. Pujol J. P. (2003). The role of reactive oxygen species in homeostasis and degradation of cartilage. Osteoarthr. Cartil. 11, 747–755. 10.1016/s1063-4584(03)00150-x 13129694

[B21] Higgins J. P. Thompson S. G. Deeks J. J. Altman D. G. (2003). Measuring inconsistency in meta-analyses. BMJ 327, 557–560. 10.1136/bmj.327.7414.557 12958120 PMC192859

[B22] Husar-Memmer E. Stadlmayr A. Datz C. Zwerina J. (2014). HFE-Related hemochromatosis: An update for the rheumatologist. Curr. Rheumatol. Rep. 16, 393. 10.1007/s11926-013-0393-4 24264720

[B23] Jing X. Lin J. Du T. Jiang Z. Li T. Wang G. (2021). Iron overload is associated with accelerated progression of osteoarthritis: The role of DMT1 mediated iron homeostasis. Front. Cell. Dev. Biol. 8, 594509. 10.3389/fcell.2020.594509 33469535 PMC7813682

[B24] Katz J. N. Arant K. R. Loeser R. F. (2021). Diagnosis and treatment of hip and knee osteoarthritis: A review. Jama 325, 568–578. 10.1001/jama.2020.22171 33560326 PMC8225295

[B25] Kennish L. Attur M. Oh C. Krasnokutsky S. Samuels J. Greenberg J. D. (2014). Age-dependent ferritin elevations and HFE C282Y mutation as risk factors for symptomatic knee osteoarthritis in males: A longitudinal cohort study. BMC Musculoskelet. Disord. 15, 8. 10.1186/1471-2474-15-8 24401005 PMC3893611

[B26] Kozijn A. E. Tartjiono M. T. Ravipati S. van der Ham F. Barrett D. A. Mastbergen S. C. (2019). Human C-reactive protein aggravates osteoarthritis development in mice on a high-fat diet. Osteoarthr. Cartil. 27, 118–128. 10.1016/j.joca.2018.09.007 30248505

[B27] Lawlor D. A. (2016). Commentary: Two-sample mendelian randomization: Opportunities and challenges. Int. J. Epidemiol. 45, 908–915. 10.1093/ije/dyw127 27427429 PMC5005949

[B28] Lee C. H. Cook S. Lee J. S. Han B. (2016). Comparison of two meta-analysis methods: Inverse-Variance-Weighted average and weighted sum of Z-scores. Genomics Inf. 14, 173–180. 10.5808/gi.2016.14.4.173 PMC5287121 28154508

[B29] Livshits G. Zhai G. Hart D. J. Kato B. S. Wang H. Williams F. M. K. (2009). Interleukin-6 is a significant predictor of radiographic knee osteoarthritis: The Chingford Study. Arthritis Rheum. 60, 2037–2045. 10.1002/art.24598 19565477 PMC2841820

[B30] Louati K. Vidal C. Berenbaum F. Sellam J. (2015). Association between diabetes mellitus and osteoarthritis: Systematic literature review and meta-analysis. RMD Open 1, e000077. 10.1136/rmdopen-2015-000077 26535137 PMC4613158

[B31] Nüesch E. Dieppe P. Reichenbach S. Williams S. Iff S. Juni P. (2011). All cause and disease specific mortality in patients with knee or hip osteoarthritis: Population based cohort study. BMJ 342, d1165. 10.1136/bmj.d1165 21385807 PMC3050438

[B32] Nugzar O. Zandman-Goddard G. Oz H. Lakstein D. Feldbrin Z. Shargorodsky M. (2018). The role of ferritin and adiponectin as predictors of cartilage damage assessed by arthroscopy in patients with symptomatic knee osteoarthritis. Best. Pract. Res. Clin. Rheumatol. 32, 662–668. 10.1016/j.berh.2019.04.004 31203924

[B33] Palmer T. M. Lawlor D. A. Harbord R. M. Sheehan N. A. Tobias J. H. Timpson N. J. (2012). Using multiple genetic variants as instrumental variables for modifiable risk factors. Stat. methods Med. Res. 21, 223–242. 10.1177/0962280210394459 21216802 PMC3917707

[B34] Palmer W. C. Vishnu P. Sanchez W. Aqel B. Riegert-Johnson D. Seaman L. A. K. (2018). Diagnosis and management of genetic iron overload disorders. J. Gen. Intern Med. 33, 2230–2236. 10.1007/s11606-018-4669-2 30225768 PMC6258594

[B35] Pilling L. C. Tamosauskaite J. Jones G. Wood A. R. Jones L. Kuo C. L. (2019). Common conditions associated with hereditary haemochromatosis genetic variants: Cohort study in UK Biobank. Bmj 364, k5222. 10.1136/bmj.k5222 30651232 PMC6334179

[B36] Qu Z. Yang F. Hong J. Wang W. Li S. Jiang G. (2020). Causal relationship of serum nutritional factors with osteoarthritis: A mendelian randomization study. Rheumatol. Oxf. 60, 2383–2390. 10.1093/rheumatology/keaa622 33167034

[B37] Sheehan N. A. Didelez V. Burton P. R. Tobin M. D. (2008). Mendelian randomisation and causal inference in observational epidemiology. PLoS Med. 5, e177. 10.1371/journal.pmed.0050177 18752343 PMC2522255

[B38] Simão M. Gavaia P. J. Camacho A. Porto G. Pinto I. J. Ea H. K. (2019). Intracellular iron uptake is favored in Hfe-KO mouse primary chondrocytes mimicking an osteoarthritis-related phenotype. Biofactors 45, 583–597. 10.1002/biof.1520 31132316

[B39] Tachmazidou I. Hatzikotoulas K. Southam L. Esparza-Gordillo J. Haberland V. Zheng J. (2019). Identification of new therapeutic targets for osteoarthritis through genome-wide analyses of UK Biobank data. Nat. Genet. 51, 230–236. 10.1038/s41588-018-0327-1 30664745 PMC6400267

[B40] Thompson J. R. Minelli C. Del Greco M. F. (2016). Mendelian randomization using public data from genetic consortia. Int. J. Biostat. 12. 10.1515/ijb-2015-0074 27092657

[B41] van Vulpen L. F. Roosendaal G. van Asbeck B. S. Mastbergen S. C. Lafeber F. P. J. G. Schutgens R. E. G. (2015). The detrimental effects of iron on the joint: A comparison between haemochromatosis and haemophilia. J. Clin. Pathol. 68, 592–600. 10.1136/jclinpath-2015-202967 25897098

[B42] Verbanck M. Chen C. Y. Neale B. Do R. (2018). Detection of widespread horizontal pleiotropy in causal relationships inferred from Mendelian randomization between complex traits and diseases. Nat. Genet. 50, 693–698. 10.1038/s41588-018-0099-7 29686387 PMC6083837

[B43] Winter W. E. Bazydlo L. A. Harris N. S. (2014). The molecular biology of human iron metabolism. Lab. Med. 45, 92–102. 10.1309/lmf28s2gimxnwhmm 24868988

[B44] Xu J. Zhang S. Tian Y. Si H. Zeng Y. Wu Y. (2022). Genetic causal association between iron status and osteoarthritis: A two-sample mendelian randomization. Nutrients 14, 3683. 10.3390/nu14183683 36145059 PMC9501024

[B45] Yang J. Zhang G. Dong D. Shang P. (2018). Effects of iron overload and oxidative damage on the musculoskeletal system in the space environment: Data from spaceflights and ground-based simulation models. Int. J. Mol. Sci. 19, 2608. 10.3390/ijms19092608 30177626 PMC6163331

[B46] Yavorska O. O. Burgess S. (2017). MendelianRandomization: an R package for performing Mendelian randomization analyses using summarized data. Int. J. Epidemiol. 46, 1734–1739. 10.1093/ije/dyx034 28398548 PMC5510723

[B47] Yazar M. Sarban S. Kocyigit A. Isikan U. E. (2005). Synovial fluid and plasma selenium, copper, zinc, and iron concentrations in patients with rheumatoid arthritis and osteoarthritis. Biol. Trace Elem. Res. 106, 123–132. 10.1385/bter:106:2:123 16116244

[B48] Zheng H. Chen C. (2015). Body mass index and risk of knee osteoarthritis: Systematic review and meta-analysis of prospective studies. BMJ Open 5, e007568. 10.1136/bmjopen-2014-007568 PMC4679914 26656979

[B49] Zhou J. Liu C. Francis M. Sun Y. Ryu M. S. Grider A. (2020). The causal effects of blood iron and copper on lipid metabolism diseases: Evidence from phenome-wide mendelian randomization study. Nutrients 12, 3174. 10.3390/nu12103174 33080795 PMC7603077

[B50] Zhou J. Liu C. Sun Y. Francis M. Ryu M. S. Grider A. (2021). Genetically predicted circulating levels of copper and zinc are associated with osteoarthritis but not with rheumatoid arthritis. Osteoarthr. Cartil. 29, 1029–1035. 10.1016/j.joca.2021.02.564 33640581

