# Supplementary material for: The effect of systemic iron status on osteoarthritis: A mendelian randomization study
Source: Front Genet. 2023 Mar 16;14:1122955. doi: 10.3389/fgene.2023.1122955 (PMC10060517; doi:10.3389/fgene.2023.1122955)
Supplement: Supplementary file 1 [file Table1.docx]

## Supplementary table 1. Associations of the instrumental SNPs with serum iron and knee osteoarthritis, hip osteoarthritis, total knee replacement, and total hip replacement.

| SNP (GRCh37 position) | Associated potential confounding factors | | Effect allele/  Other allele | Effect allele frequency | SNP & serum iron associations |  | SNP & osteoarthritis associations | | | | | | | |
| --- | --- | --- | --- | --- | --- | --- | --- | --- | --- | --- | --- | --- | --- | --- |
|  |  |  |  |  | Beta (SE) |  | Knee osteoarthritis | | Hip osteoarthritis | | Total knee replacement | | Total hip replacement | |
|  |  |  |  |  |  |  | Beta (SE) | *P* | Beta (SE) | *P* | Beta (SE) | *P* | Beta (SE) | *P* |
| rs2228145 (1:154426970) | interleukin 6; C-reactive protein | | C/A | 0.407 | 0.026 (0.004) |  | -0.005 (0.007) | 0.535 | 0.010 (0.009) | 0.283 | -0.005 (0.012) | 0.659 | 0.019 (0.011) | 0.092 |
| rs35945185 (1:66137239) |  | | A/G | 0.365 | 0.031 (0.004) |  | 0.009 (0.011) | 0.408 | 0.013 (0.013) | 0.338 | 0.005 (0.015) | 0.762 | 0.016 (0.014) | 0.274 |
| rs57659670 (15:45398438) |  | | C/T | 0.075 | -0.042 (0.007) |  | 0.036(0.013) | 0.007 | 0.036 (0.017) | 0.037 | 0.049 (0.023) | 0.035 | 0.019 (0.021) | 0.372 |
| rs77262773 (17:67249711) |  | | T/C | 0.026 | 0.081 (0.014) |  | 0.009 (0.025) | 0.738 | 0.047 (0.032) | 0.145 | 0.017 (0.04) | 0.680 | 0.033 (0.037) | 0.370 |
| rs2005682 (19:35947661) |  | | T/A | 0.305 | -0.029 (0.004) |  | 0.004 (0.008) | 0.597 | -0.004 (0.010) | 0.656 | -0.007 (0.013) | 0.600 | -0.015 (0.012) | 0.214 |
| rs13007705 (2:239069196) |  | | T/C | 0.425 | 0.029 (0.004) |  | 0.010 (0.007) | 0.165 | 0.005 (0.009) | 0.602 | 0.007 (0.012) | 0.599 | -0.001 (0.011) | 0.917 |
| rs855791 (22:37462936) | glycosylated haemoglobin | | A/G | 0.431 | -0.170 (0.005) |  | -0.006 (0.007) | 0.403 | -0.012 (0.009) | 0.205 | -0.008 (0.013) | 0.512 | -0.013 (0.011) | 0.276 |
| rs748587164 (3:133476750)^*^ |  | | A/T | 0.001 | -0.520 (0.069) |  | - | - | - | - | - | - | - | - |
| rs4854760 (3:133498741) |  | | G/A | 0.313 | 0.053 (0.005) |  | 0.001 (0.007) | 0.933 | 0.001 (0.009) | 0.947 | -0.003 (0.013) | 0.831 | 0.014 (0.012) | 0.236 |
| rs7630745 (3:66427029) |  | | C/T | 0.358 | 0.025 (0.004) |  | 0.001 (0.008) | 0.949 | -0.014 (0.009) | 0.144 | 0.026 (0.013) | 0.044 | -0.009 (0.011) | 0.434 |
| rs9399136 (6:135402339) intergenic | self-reported high cholesterol | | C/T | 0.259 | 0.057 (0.004) |  | -0.018 (0.008) | 0.031 | 0.005 (0.011) | 0.633 | -0.023 (0.014) | 0.101 | 0.009 (0.013) | 0.484 |
| rs1799945 (6:26091179) | glycosylated haemoglobin | | G/C | 0.137 | 0.170 (0.005) |  | 0.007 (0.010) | 0.515 | 0.013 (0.013) | 0.303 | -0.011 (0.017) | 0.513 | 0.017 (0.016) | 0.284 |
| rs1800562 (6:26093141) | glycosylated haemoglobin; low-density lipoprotein; total cholesterol | | A/G | 0.068 | 0.270 (0.008) |  | 0.034 (0.014) | 0.019 | 0.105 (0.018) | 2.9×10^-9^ | 0.036 (0.024) | 0.132 | 0.101 (0.021) | 1.2×10^-6^ |
| rs7385804 (7:100235970) |  | | C/A | 0.371 | -0.057 (0.004) |  | -0.007 (0.007) | 0.327 | -0.020 (0.009) | 0.032 | 0.002 (0.012) | 0.865 | -0.020 (0.011) | 0.084 |
| rs12718598 (7:50428445) |  | | C/T | 0.463 | 0.027 (0.004) |  | 0.002 (0.007) | 0.790 | -2×10^-4^ (0.012) | 0.987 | 0.006 (0.012) | 0.617 | 0.001 (0.011) | 0.934 |
| SNP, single nucleotide polymorphism; SE, standard error. | | | | | | | | | | | | | | |
|  | |  |  |  |  |  |  |  |  |  |  |  |  |  |

## Supplementary table 2. Associations of the instrumental SNPs with transferrin saturation and knee osteoarthritis, hip osteoarthritis, total knee replacement, and total hip replacement.

| SNP (GRCh37 position) | Associated potential confounding factors | Effect allele/  Other allele | Effect allele frequency | SNP & serum iron associations |  | SNP & osteoarthritis associations | | | | | | | |
| --- | --- | --- | --- | --- | --- | --- | --- | --- | --- | --- | --- | --- | --- |
|  |  |  |  | Beta (SE) |  | Knee osteoarthritis | | Hip osteoarthritis | | Total knee replacement | | Total hip replacement | |
|  |  |  |  |  |  | Beta (SE) | *P* | Beta (SE) | *P* | Beta (SE) | *P* | Beta (SE) | *P* |
| rs57659670 (15:45398438) |  | C/T | 0.425 | -0.058 (0.008) |  | 0.036 (0.013) | 0.007 | 0.036 (0.017) | 0.037 | 0.049 (0.023) | 0.035 | 0.019 (0.021) | 0.372 |
| rs2005682 (19:35947661) |  | T/A | 0.259 | -0.032 (0.005) |  | 0.004 (0.008) | 0.597 | -0.004 (0.010) | 0.656 | -0.007 (0.013) | 0.600 | -0.015 (0.012) | 0.214 |
| rs13007705 (2:239069196) |  | T/C | 0.075 | 0.033 (0.005) |  | 0.010 (0.007) | 0.165 | 0.005 (0.009) | 0.602 | 0.007 (0.012) | 0.599 | -0.001 (0.011) | 0.917 |
| rs855791 (22:37462936) | glycosylated haemoglobin | A/G | 0.305 | -0.170 (0.005) |  | -0.006 (0.007) | 0.403 | -0.012 (0.009) | 0.205 | -0.008 (0.013) | 0.512 | -0.013 (0.011) | 0.276 |
| rs748587164 (3:133476750)^*^ |  | A/T | 0.001 | 0.720 (0.084) |  | - | - | - | - | - | - | - | - |
| rs4854760 (3:133498741) |  | G/A | 0.313 | -0.096 (0.006) |  | 0.001 (0.007) | 0.933 | 0.001 (0.009) | 0.947 | -0.003 (0.013) | 0.831 | 0.014 (0.012) | 0.236 |
| rs3817672 (3:195800811) |  | C/T | 0.440 | 0.026 (0.004) |  | -0.003 (0.007) | 0.651 | 0.006 (0.009) | 0.52 | -0.002 (0.013) | 0.898 | 0.010 (0.011) | 0.348 |
| rs9399136 (6:135402339) | self-reported high cholesterol | C/T | 0.137 | 0.067 (0.005) |  | -0.018 (0.008) | 0.031 | 0.005 (0.011) | 0.633 | -0.023 (0.014) | 0.101 | 0.009 (0.013) | 0.484 |
| rs1799945 (6:26091179) | glycosylated haemoglobin | G/C | 0.068 | 0.210 (0.008) |  | 0.007 (0.010) | 0.515 | 0.013 (0.013) | 0.303 | -0.011 (0.017) | 0.513 | 0.017 (0.016) | 0.284 |
| rs1800562 (6:26093141) | glycosylated haemoglobin; low-density lipoprotein; total cholesterol | A/G | 0.371 | 0.450 (0.013) |  | 0.034 (0.014) | 0.019 | 0.105 (0.018) | 2.9×10^-9^ | 0.036 (0.024) | 0.132 | 0.101 (0.021) | 1.2×10^-6^ |
| rs7385804 (7:100235970) |  | C/A | 0.431 | -0.062 (0.005) |  | -0.007 (0.007) | 0.327 | -0.020 (0.009) | 0.032 | 0.002 (0.012) | 0.865 | -0.020 (0.011) | 0.084 |
| rs748587164 is not in 1000G reference panel.  SNP, single nucleotide polymorphism; SE, standard error. | | | | | | | | | | | | | |

## Supplementary table 3. Associations of the instrumental SNPs with ferritin and knee osteoarthritis, hip osteoarthritis, total knee replacement, and total hip replacement.

| SNP (GRCh37 position) | Associated potential confounding factors | Effect allele/  Other allele | Effect allele frequency | SNP & serum iron associations |  | SNP & osteoarthritis associations | | | | | | | |  |  |
| --- | --- | --- | --- | --- | --- | --- | --- | --- | --- | --- | --- | --- | --- | --- | --- |
|  |  |  |  | Beta (SE) |  | Knee osteoarthritis | | Hip osteoarthritis | | Total knee replacement | | Total hip replacement | |  |  |
|  |  |  |  |  |  | Beta (SE) | *P* | Beta (SE) | *P* | Beta (SE) | *P* | Beta (SE) | *P* |  |  |
| rs10801913 (1:116214279) |  | A/G | 0.021 | 0.024 (0.004) |  | 0.004 (0.008) | 0.609 | 0.008 (0.010) | 0.393 | 0.015 (0.013) | 0.258 | 0.024 (0.012) | 0.047 |  |  |
| rs6025 (1:169519049) |  | T/C | 0.307 | 0.150 (0.013) |  | 0.026 (0.024) | 0.279 | -0.078 (0.031) | 0.011 | 0.004 (0.041) | 0.919 | -0.110 (0.037) | 0.003 |  |  |
| rs551459670 (1:220288690) |  | A/G | 0.028 | 0.140 (0.020) |  | -0.081 (0.061) | 0.181 | -0.041 (0.078) | 0.601 | -0.065 (0.089) | 0.464 | -0.001 (0.085) | 0.99 |  |  |
| rs75965181 (1:22584002) | bone mineral density | A/T | 0.011 | -0.120 (0.011) |  | -0.036 (0.027) | 0.191 | -0.027 (0.034) | 0.421 | -0.014 (0.046) | 0.766 | -0.022 (0.042) | 0.598 |  |  |
| rs17476364 (10:71094504) | glycosylated haemoglobin; self-reported high cholesterol | C/T | 0.368 | 0.043 (0.006) |  | 0.006 (0.012) | 0.629 | 0.002 (0.015) | 0.897 | -0.003 (0.02) | 0.873 | 0.023 (0.018) | 0.202 |  |  |
| rs12419620 (11:2232553) |  | G/T | 0.274 | -0.031 (0.005) |  | 0.002 (0.01) | 0.863 | 0.024 (0.012) | 0.058 | -0.008 (0.017) | 0.635 | 0.023 (0.015) | 0.126 |  |  |
| rs12807014 (11:47760078) | trunk fat mass; waist circumference; body mass index | C/T | 0.288 | -0.029 (0.004) |  | -0.016 (0.008) | 0.049 | -0.019 (0.010) | 0.062 | -0.017 (0.014) | 0.214 | -0.023 (0.012) | 0.058 |  |  |
| rs4938939 (11:60160838) |  | A/G | 0.002 | 0.022 (0.004) |  | 0.005 (0.008) | 0.490 | -0.006 (0.010) | 0.563 | 0.005 (0.013) | 0.717 | -0.004 (0.012) | 0.721 |  |  |
| rs996347 (14:34410892) |  | C/T | 0.003 | 0.049 (0.004) |  | 0.002 (0.007) | 0.751 | 0.007 (0.009) | 0.437 | -0.014 (0.013) | 0.257 | 0.016 (0.012) | 0.174 |  |  |
| rs57659670 (15:45398438) |  | C/T | 0.005 | -0.140 (0.008) |  | 0.036 (0.013) | 0.007 | 0.036 (0.017) | 0.037 | 0.049 (0.023) | 0.035 | 0.019 (0.021) | 0.372 |  |  |
| rs3743171 (15:65916527) | body mass index; bone mineral density | T/A | 0.003 | -0.024 (0.004) |  | -0.020 (0.009) | 0.024 | -0.002 (0.011) | 0.888 | -0.021 (0.015) | 0.169 | -0.009 (0.014) | 0.515 |  |  |
| rs9921222 (16:375782) | bone mineral density; trunk fat-free mass | C/T | 0.112 | 0.025 (0.004) |  | 0.005 (0.007) | 0.479 | 0.010 (0.009) | 0.292 | 0.001 (0.013) | 0.964 | 0.015 (0.011) | 0.186 |  |  |
| rs3747602 (16:4802386) |  | G/T | 0.126 | 0.021 (0.004) |  | -0.008 (0.007) | 0.276 | -0.022 (0.009) | 0.019 | -0.018 (0.013) | 0.157 | -0.027 (0.011) | 0.020 |  |  |
| rs34523089 (17:56436109) |  | T/C | 0.446 | 0.069 (0.005) |  | -0.009 (0.010) | 0.350 | -0.001 (0.012) | 0.939 | -0.014 (0.017) | 0.395 | -0.006 (0.015) | 0.700 |  |  |
| rs535064984 (17:7020297) | self-reported high cholesterol | C/T | 0.079 | 0.230(0.026) |  | -0.030 (0.055) | 0.589 | 0.060 (0.069) | 0.380 | 0.132 (0.086) | 0.123 | 0.031(0.08) | 0.702 |  |  |
| rs1542752 (17:72938100) |  | T/C | 0.323 | 0.034 (0.005) |  | 0.005 (0.01) | 0.649 | 0.020 (0.012) | 0.118 | 0.017 (0.017) | 0.306 | 0.025 (0.015) | 0.098 |  |  |
| rs55789050 (17:9793417) | trunk fat mass; body fat mass | T/A | 0.479 | -0.027 (0.004) |  | -0.003 (0.008) | 0.728 | -0.002 (0.010) | 0.852 | -0.020 (0.013) | 0.132 | -0.006 (0.012) | 0.586 |  |  |
| rs4808802 (19:18577873) | total cholesterol | C/G | 0.360 | 0.028 (0.004) |  | -0.003 (0.009) | 0.728 | -0.010 (0.011) | 0.385 | -0.009 (0.015) | 0.562 | -0.001 (0.014) | 0.918 |  |  |
| rs601338 (19:49206674) | self-reported high cholesterol; total cholesterol; low density lipoprotein | G/A | 0.108 | 0.028 (0.004) |  | 0.015 (0.007) | 0.039 | 0.036 (0.009) | 7.6×10^-5^ | -0.009 (0.012) | 0.472 | 0.038(0.011) | 4.9×10^-4^ |  |  |
| rs143041401 (19:49550116) |  | A/G | 0.161 | 0.110 (0.013) |  | -0.029 (0.037) | 0.438 | -0.051 (0.043) | 0.240 | 0.076 (0.057) | 0.177 | -0.067 (0.051) | 0.187 |  |  |
| rs708686 (19:5840619) |  | T/C | 0.274 | -0.031 (0.004) |  | 0.010 (0.008) | 0.232 | -0.004 (0.010) | 0.726 | 0.035 (0.014) | 0.011 | 0.002 (0.012) | 0.885 |  |  |
| rs12693541 (2:190418690) |  | C/T | 0.293 | 0.079 (0.005) |  | -0.014 (0.011) | 0.181 | -0.017 (0.014) | 0.234 | -0.026 (0.019) | 0.164 | -0.021 (0.017) | 0.226 |  |  |
| rs1250259 (2:216300482) | low-density lipoprotein; total cholesterol | T/A | 0.007 | -0.024 (0.004) |  | -0.002 (0.008) | 0.761 | -0.007 (0.01) | 0.471 | -0.017 (0.014) | 0.227 | -0.015 (0.012) | 0.213 |  |  |
| rs1260326 (2:27730940) | triglycerides; C reactive protein; trunk fat-free mass; total cholesterol; self-reported high cholesterol | T/C | 0.355 | 0.025 (0.004) |  | -0.007 (0.007) | 0.351 | -0.026 (0.009) | 0.006 | -0.031 (0.012) | 0.014 | -0.032 (0.011) | 0.004 |  |  |
| rs6757653 (2:29171804) |  | T/C | 0.075 | 0.032 (0.004) |  | 0.016 (0.008) | 0.043 | 0.019 (0.010) | 0.052 | -0.014 (0.013) | 0.286 | 0.018 (0.012) | 0.140 |  |  |
| rs6029148 (20:39124408) |  | A/G | 0.191 | 0.046 (0.006) |  | 0.012 (0.014) | 0.385 | 0.051 (0.018) | 0.004 | 0.060 (0.024) | 0.011 | 0.058 (0.021) | 0.006 |  |  |
| rs855791 (22:37462936) | glycosylated haemoglobin | A/G | 0.492 | -0.044 (0.003) |  | -0.006 (0.007) | 0.403 | -0.012 (0.009) | 0.205 | -0.008 (0.013) | 0.512 | -0.013 (0.011) | 0.276 |  |  |
| rs1131262 (3:133941320) |  | T/C | 0.368 | -0.032 (0.005) |  | -0.014 (0.011) | 0.185 | -0.018 (0.014) | 0.203 | -0.013 (0.018) | 0.489 | -0.046 (0.017) | 0.007 |  |  |
| rs762752083 (3:52536039)^*^ |  | T/G | 0.006 | 0.350 (0.046) |  | - | - | - | - | - | - | - | - |  |  |
| rs750717575 (3:52536725)^*^ |  | A/G | 0.333 | 0.240 (0.041) |  | - | - | - | - | - | - | - | - |  |  |
| rs745795585 (3:52539395)^*^ |  | A/G | 0.153 | 0.290 (0.031) |  | - | - | - | - | - | - | - | - |  |  |
| rs34216132 (3:52727675) |  | C/G | 0.258 | 0.170 (0.028) |  | 0.073 (0.07) | 0.297 | 0.093 (0.084) | 0.268 | 0.133 (0.110) | 0.227 | 0.136 (0.101) | 0.180 |  |  |
| rs1799945 (6:26091179) | glycosylated haemoglobin | G/C | 0.218 | 0.059 (0.005) |  | 0.007 (0.01) | 0.515 | 0.013 (0.013) | 0.303 | -0.011 (0.017) | 0.513 | 0.017 (0.016) | 0.284 |  |  |
| rs1800562 (6:26093141) | glycosylated haemoglobin; low-density lipoprotein; total cholesterol | A/G | 0.484 | 0.130 (0.008) |  | 0.034 (0.014) | 0.019 | 0.105 (0.018) | 2.9×10^-9^ | 0.036 (0.024) | 0.132 | 0.101 (0.021) | 1.2×10^-6^ |  |  |
| rs36184164 (6:43781092) |  | G/T | 0.016 | 0.036 (0.005) |  | 0.001 (0.013) | 0.925 | -0.006 (0.016) | 0.695 | -0.007 (0.019) | 0.705 | -0.007 (0.017) | 0.691 |  |  |
| rs2529440 (7:30511794) |  | T/C | 0.071 | -0.035 (0.003) |  | 0.002 (0.007) | 0.794 | 0.010 (0.009) | 0.291 | 0.009 (0.012) | 0.486 | 0.008 (0.011) | 0.498 |  |  |
| rs4841429 (8:10568529) | bone mineral density | G/A | 0.119 | 0.060 (0.006) |  | -0.009 (0.013) | 0.527 | -0.011 (0.017) | 0.544 | -0.017 (0.023) | 0.475 | -0.012 (0.021) | 0.552 |  |  |
| rs2954029 (8:126490972) | triglycerides; total cholesterol; low-density lipoprotein; high-density lipoprotein; trunk fat mass | T/A | 0.137 | -0.024 (0.003) |  | -0.004 (0.007) | 0.555 | -0.011 (0.009) | 0.214 | 0.006 (0.012) | 0.64 | -0.010 (0.011) | 0.369 |  |  |
| rs13253974 (8:23377910) |  | A/G | 0.068 | 0.024 (0.004) |  | -0.002 (0.008) | 0.833 | -0.007 (0.01) | 0.477 | 0.028 (0.013) | 0.033 | -0.011 (0.012) | 0.33 |  |  |
| rs7865362 (9:33117965) |  | T/C | 0.161 | 0.025 (0.004) |  | -0.001 (0.007) | 0.913 | 0.021 (0.009) | 0.027 | -0.003 (0.013) | 0.819 | 0.022 (0.011) | 0.057 |  |  |
| rs745795585, rs762752083, and rs750717575 are not in 1000G reference panel.  SNP, single nucleotide polymorphism; SE, standard error. | | | | | | | | | | | | | |  | 0.431 |

## Supplementary table 4. Associations of the instrumental SNPs with total iron-binding capacity and knee osteoarthritis, hip osteoarthritis, total knee replacement, and total hip replacement.

| SNP (GRCh37 position) | Associated potential confounding factors^*^ | Effect allele/  Other allele | Effect allele frequency | SNP & serum iron associations^*^ |  | SNP & osteoarthritis associations | | | | | | | |  |  |
| --- | --- | --- | --- | --- | --- | --- | --- | --- | --- | --- | --- | --- | --- | --- | --- |
|  |  |  |  | Beta (SE) |  | Knee osteoarthritis | | Hip osteoarthritis | | Total knee replacement | | Total hip replacement | |  |  |
|  |  |  |  |  |  | Beta (SE) | *P* | Beta (SE) | *P* | Beta (SE) | *P* | Beta (SE) | *P* |  |  |
| rs6025 (1:169519049) |  | T/C | 0.028 | -0.093 (0.018) |  | 0.026 (0.024) | 0.279 | -0.078 (0.031) | 0.011 | 0.004 (0.041) | 0.919 | -0.110 (0.037) | 0.003 |  |  |
| rs174546 (11:61569830) |  | T/C | 0.335 | 0.046 (0.005) |  | -0.008 (0.007) | 0.267 | -0.012 (0.009) | 0.21 | -0.001 (0.012) | 0.934 | 0.007 (0.011) | 0.531 |  |  |
| rs17580 (14:94847262) |  | A/T | 0.259 | 0.076 (0.012) |  | -0.013 (0.019) | 0.495 | -0.034 (0.025) | 0.172 | -0.065 (0.031) | 0.039 | -0.038 (0.028) | 0.18 |  |  |
| rs57659670 (15:45398438) |  | C/T | 0.038 | 0.077 (0.009) |  | 0.036 (0.013) | 0.007 | 0.036 (0.017) | 0.037 | 0.049 (0.023) | 0.035 | 0.019 (0.021) | 0.372 |  |  |
| rs112727702 (19:50091204) |  | T/G | 0.075 | 0.043 (0.006) |  | -0.007 (0.011) | 0.531 | -0.029 (0.014) | 0.035 | -0.006 (0.017) | 0.715 | -0.037 (0.015) | 0.015 |  |  |
| rs12693541 (2:190418690) |  | C/T | 0.232 | -0.048 (0.007) |  | -0.014 (0.011) | 0.181 | -0.017 (0.014) | 0.234 | -0.026 (0.019) | 0.164 | -0.021 (0.017) | 0.226 |  |  |
| rs1132274 (20:17596155) |  | A/C | 0.168 | 0.036 (0.006) |  | 0.015 (0.01) | 0.145 | 0.009 (0.013) | 0.508 | 0.028 (0.017) | 0.104 | -0.001 (0.016) | 0.965 |  |  |
| rs855791 (22:37462936) | glycosylated haemoglobin | A/G | 0.119 | 0.026 (0.005) |  | -0.006 (0.007) | 0.403 | -0.012 (0.009) | 0.205 | -0.008 (0.013) | 0.512 | -0.013 (0.011) | 0.276 |  |  |
| rs4854760 (3:133498741) |  | G/A | 0.001 | 0.340 (0.008) |  | 0.001 (0.007) | 0.933 | 0.001 (0.009) | 0.947 | -0.003 (0.013) | 0.831 | 0.014 (0.012) | 0.236 |  |  |
| rs3817672 (3:195800811) |  | C/T | 0.313 | -0.031 (0.005) |  | -0.003 (0.007) | 0.651 | 0.006 (0.009) | 0.52 | -0.002 (0.013) | 0.898 | 0.010 (0.011) | 0.348 |  |  |
| rs59950280 (4:3452345) |  | A/G | 0.440 | 0.033 (0.005) |  | -0.011 (0.008) | 0.182 | -0.019 (0.01) | 0.05 | -0.037 (0.013) | 0.005 | -0.027 (0.012) | 0.022 |  |  |
| rs9399136 (6:135402339) | self-reported high cholesterol | C/T | 0.137 | -0.033 (0.005) |  | -0.018 (0.008) | 0.031 | 0.005 (0.011) | 0.633 | -0.023 (0.014) | 0.101 | 0.009 (0.013) | 0.484 |  |  |
| rs1799945 (6:26091179) | glycosylated haemoglobin | G/C | 0.068 | -0.120 (0.008) |  | 0.007 (0.01) | 0.515 | 0.013 (0.013) | 0.303 | -0.011 (0.017) | 0.513 | 0.017 (0.016) | 0.284 |  |  |
| rs1800562 (6:26093141) | glycosylated haemoglobin; low-density lipoprotein; total cholesterol | A/G | 0.214 | -0.450 (0.010) |  | 0.034 (0.014) | 0.019 | 0.105 (0.018) | 2.9×10^-9^ | 0.036 (0.024) | 0.132 | 0.101 (0.021) | 1.2×10^-6^ |  |  |
| rs1495743 (8:18273300) |  | G/C | 0.354 | -0.043 (0.006) |  | -1×10^-4^ (0.015) | 0.078 | -0.019 (0.011) | 0.078 | -0.020 (0.015) | 0.160 | -0.032 (0.013) | 0.017 |  |  |
| SNP, single nucleotide polymorphism; SE, standard error. | | | | | | | | | | | | | |  | 0.431 |

## Supplementary table 5. Leave-one-out analysis of the causal association between the four systemic iron status biomarkers with conservative instruments and hip osteoarthritis & total hip replacement.

| Iron biomarkers | SNP (leave out) | Hip osteoarthritis | | Total hip replacement | |  |
| --- | --- | --- | --- | --- | --- | --- |
|  |  | OR (95% CI) | *P* value^*^ | OR (95% CI) | *P* value^*^ |  |
| Serum iron | rs1800562 | 1.06 (0.98, 1.16) | 0.154 | 1.08 (0.97, 1.20) | 0.159 |  |
|  | rs855791 | 1.27 (1.15, 1.40) | **<0.001** | 1.28 (1.14, 1.44) | **<0.001** |  |
|  | rs1799945 | 1.20 (1.11, 1.30) | **<0.001** | 1.22 (1.10, 1.34) | **<0.001** |  |
|  | rs57659670 | 1.18 (1.10, 1.27) | **<0.001** | 1.20 (1.10, 1.30) | **<0.001** |  |
| Transferrin saturation | rs1800562 | 1.06 (0.98, 1.14) | 0.183 | 1.07 (0.97, 1.18) | 0.164 |  |
|  | rs855791 | 1.19 (1.12, 1.27) | **<0.001** | 1.19 (1.11, 1.29) | **<0.001** |  |
|  | rs1799945 | 1.18 (1.11, 1.26) | **<0.001** | 1.19 (1.10, 1.28) | **<0.001** |  |
|  | rs57659670 | 1.17 (1.10, 1.23) | **<0.001** | 1.17 (1.09, 1.25) | **<0.001** |  |
| Ferritin | rs1800562 | 0.94 (0.78, 1.14) | 0.544 | 1.03 (0.82, 1.30) | 0.791 |  |
|  | rs855791 | 1.25 (1.06, 1.47) | **0.009** | 1.34 (1.10, 1.63) | **0.004** |  |
|  | rs1799945 | 1.25 (1.07, 1.48) | **0.006** | 1.34 (1.10, 1.63) | **0.004** |  |
|  | rs57659670 | 1.75 (1.43, 2.13) | **<0.001** | 1.76 (1.39, 2.23) | **<0.001** |  |
| Total iron-binding capacity | rs1800562 | 0.97 (0.81, 1.16) | 0.727 | 0.91 (0.73, 1.13) | 0.399 |  |
|  | rs855791 | 0.82 (0.76, 0.88) | **<0.001** | 0.82 (0.75, 0.89) | **<0.001** |  |
|  | rs1799945 | 0.81 (0.75, 0.87) | **<0.001** | 0.81 (0.74, 0.88) | **<0.001** |  |
|  | rs57659670 | 0.80 (0.75, 0.86) | **<0.001** | 0.80 (0.74, 0.88) | **<0.001** |  |
| * Bolded *P* values indicate statistical significance (<0.012).  IVW, inverse variance weighted; OR, odds ratio; CI: compatibility/confidence interval. | | | | | |  |
| Supplementary table 6. Associations of the instrumental SNPs with four systemic iron status biomarkers and knee osteoarthritis and hip osteoarthritis in the 2019 osteoarthritis genome-wide association study dataset.  \| Iron biomarkers \| Instrument set (number of instruments) \| Analysis method \| Knee osteoarthritis \| \| Hip osteoarthritis \| \| \| --- \| --- \| --- \| --- \| --- \| --- \| --- \| \| OR (95% CI) \| *P* value^*^ \| OR (95% CI) \| *P* value^*^ \| \| Serum iron \| Liberal instruments (14) \| IVW \| 1.04 (0.96, 1.13) \| 0.309 \| 1.15 (1.01, 1.30) \| **0.037** \| \| Weighted median \| 1.06 (0.97, 1.16) \| 0.174 \| 1.04 (0.91, 1.19) \| 0.533 \| \| MR-Egger \| 1.06 (0.95, 1.20) \| 0.298 \| 1.22 (1.01, 1.47) \| **0.042** \| \| Sensitivity instruments (12) \| IVW \| 1.07 (0.99, 1.16) \| 0.059 \| 1.15 (1.00, 1.32) \| **0.049** \| \| Weighted median \| 1.07 (0.98, 1.16) \| 0.148 \| 1.04 (0.91, 1.19) \| 0.515 \| \| MR-Egger \| 1.11 (0.99, 1.24) \| 0.065 \| 1.23 (1.00, 1.51) \| **0.049** \| \| Conservative instruments (4) \| IVW \| 1.09 (0.98, 1.22) \| 0.115 \| 1.15 (0.90, 1.47) \| 0.248 \| \| Weighted median \| 1.07 (0.98, 1.16) \| 0.111 \| 1.04 (0.91, 1.19) \| 0.538 \| \| Transferrin saturation \| Liberal instruments (10) \| IVW \| 1.05 (0.98, 1.12) \| 0.175 \| 1.15 (1.05, 1.26) \| **0.002** \| \| Weighted median \| 1.09 (1.02, 1.16) \| **0.010** \| 1.14 (1.02, 1.29) \| **0.023** \| \| MR-Egger \| 1.07 (0.97, 1.18) \| 0.194 \| 1.20 (1.06, 1.37) \| **0.005** \| \| Sensitivity instruments (8) \| IVW \| 1.05 (0.99, 1.12) \| 0.096 \| 1.16 (1.05, 1.27) \| **0.002** \| \| Weighted median \| 1.09 (1.02, 1.16) \| **0.010** \| 1.15 (1.02, 1.30) \| **0.018** \| \| MR-Egger \| 1.06 (0.96, 1.17) \| 0.225 \| 1.20 (1.05, 1.37) \| **0.009** \| \| Conservative instruments (4) \| IVW \| 1.05 (0.95, 1.16) \| 0.366 \| 1.15 (0.99, 1.35) \| 0.075 \| \| Weighted median \| 1.07 (1.01, 1.14) \| 0.034 \| 1.13 (1.03, 1.25) \| **0.013** \| \| Ferritin \| Liberal instruments (37) \| IVW \| 0.96 (0.86, 1.08) \| 0.495 \| 1.12 (0.94, 1.33) \| 0.189 \| \| Weighted median \| 0.90 (0.77, 1.05) \| 0.172 \| 1.11 (0.92, 1.32) \| 0.275 \| \| MR-Egger \| 0.89 (0.72, 1.09) \| 0.258 \| 1.16 (0.84, 1.60) \| 0.362 \| \| Sensitivity instruments (33) \| IVW \| 0.92 (0.81, 1.06) \| 0.241 \| 1.08 (0.88, 1.32) \| 0.467 \| \| Weighted median \| 0.87 (0.73, 1.02) \| 0.086 \| 0.99 (0.82, 1.22) \| 0.992 \| \| MR-Egger \| 0.92 (0.71, 1.18) \| 0.504 \| 1.27 (0.88, 1.83) \| 0.208 \| \| Conservative instruments (4) \| IVW \| 0.99 (0.72, 1.36) \| 0.951 \| 1.32 (0.77, 2.27) \| 0.308 \| \| Weighted median \| 0.99 (0.79, 1.27) \| 0.985 \| 0.98 (0.73, 1.33) \| 0.911 \| \| Total iron-binding capacity \| Liberal instruments (15) \| IVW \| 0.96 (0.91, 1.02) \| 0.201 \| 0.90 (0.82, 0.98) \| **0.013** \| \| Weighted median \| 0.96 (0.91, 1.00) \| 0.074 \| 0.94 (0.86, 1.03) \| 0.181 \| \| MR-Egger \| 0.95 (0.88, 1.02) \| 0.150 \| 0.88 (0.79, 0.99) \| **0.031** \| \| Sensitivity instruments (33) \| IVW \| 0.96 (0.91, 1.02) \| 0.164 \| 0.90 (0.82, 0.98) \| **0.015** \| \| Weighted median \| 0.96 (0.91, 1.00) \| 0.071 \| 0.94 (0.86, 1.03) \| 0.179 \| \| MR-Egger \| 0.96 (0.89, 1.03) \| 0.202 \| 0.89 (0.79, 0.99) \| **0.043** \| \| Conservative instruments (4) \| IVW \| 0.95 (0.83, 1.08) \| 0.402 \| 0.79 (0.71, 0.88) \| **<0.001** \| \| Weighted median \| 0.94 (0.87, 1.01) \| 0.076 \| 0.79 (0.72, 0.86) \| **<0.001** \| \| * Bolded *P* values indicate value of <0.05.  IVW, inverse variance weighted; OR, odds ratio; CI: compatibility/confidence interval. \| \| \| \| \| \| \| | | | | | | |
